# Transcatheter pulmonary valve implantation in clinical practice: A nationwide survey of cardiological implanting and non-implanting physicians

**DOI:** 10.1016/j.ijcchd.2023.100478

**Published:** 2023-10-05

**Authors:** Biagio Castaldi, Gianfranco Butera, Massimo Chessa, Lorenzo Galletti, Alessandro Giamberti, Luca Giugno, Aurelio Secinaro, Vladimiro Vida, Giovanni Di Salvo, Mario Carminati

**Affiliations:** a pediatric Cardiology Unit, Department of Women's and Children's Health, University of Padua, Italy; b Cardiology, Cardiac Surgery and Heart Lung Transplantation, ERN GUARD-Heart, Bambino Gesù Hospital and Research Institute, IRCCS, Rome, Italy; c Adult Congenital Heart Disease Cardiology Unit - Pediatric and Adult Congenital Heart Centre, IRCCS Policlinico San Donato, San Donato Milanese, Milan, Italy; d San Raffaele Vita e Salute University, Milan, Italy; e Congenital Cardiac Surgery Unit, IRCCS Policlinico San Donato, San Donato Milanese, Milan, Italy; f Department of Pediatric and Adult Congenital Cardiology, IRCCS Policlinico San Donato, San Donato Milanese, Milan, Italy; g Advanced Cardiothoracic Imaging Unit, Bambino Gesù Hospital and Research Institute, IRCCS, Rome, Italy; h Pediatric Cardiac Surgery Unit, Department of Cardiac, Thoracic, Vascular Sciences and Public Health, University of Padua, Italy

**Keywords:** TPVI, PVR, RVOT dysfunction, Survey

## Abstract

**Aim:**

Transcatheter Pulmonary Valve Implantation (TPVI), when feasible, is the first-line approach to pulmonary valve replacement. Our aim was to obtain a picture of current TPVI practice in Italy.

**Methods:**

After conducting a literature review on TPVI, online surveys were devised by an Advisory Board of 10 experts from the three Italian reference centers for congenital heart diseases and sent electronically to physicians working either in implanting center or in referral non-implanting cardiologic centers.

**Results:**

Approximately 450 physicians across Italy were invited to contribute. 82 (18%) physicians answered. EchoColorDoppler, electrocardiogram and cardiac magnetic resonance were considered the first line approach to monitor these patients, before and after TPVI.

For non-implanting centers, reasons for non-referral of patients for PVR were: paucisymptomatic disease (67%) and patients’ poor adherence to disease management programs (41%), but also the lack of connections with specialized centers (33%). For implanters, the main reasons for refraining from TPVI were: high risk for coronary compression (67% first rank), the need for concomitant cardiac surgical procedures (39% first rank) and the unsuitable anatomy of the conduit (39% first rank). The availability of new larger valves of a self-expandable nature was indicated as a key technological development for expanding the cohort of patients currently eligible for TPVI.

**Conclusions:**

Despite a non-invasive imaging protocol for the follow up and selection of patients candidate to TPVI is well implemented in Italy, there is still a lack in connections between non-implanting and implanting centers.

## Highlights

• Non-implanters perceive TPVI it as a niche procedure, to be reserved for symptomaticcases with a high surgical risk.
• The lack of an official hub-and-spoke network, and a clear identification of ACHD referralcenter, may limit the access to TPVI.
• Non-implanters should be periodically updated on new tools for the patients’ selection and on the new devices available.

## 1 Introduction

Transcatheter pulmonary valve implantation (TPVI) is a minimally invasive alternative to surgery for earlier treatment of a dysfunctional right ventricular outflow tract (RVOT) [1]. This technique is now considered the most appropriate first-line approach to pulmonary valve replacement (PVR) in selected patients [1,2].

Even though the number of patients undergoing TPVI has been steadily increasing [3], previously published data suggest that only a minority of patients theoretically eligible for TPVI actually receive the treatment [4,5]. Moreover, the cohort of patients currently eligible for TPVI is a relatively small percentage of patients (∼20–25%) with indication for RVOT reconstruction and PVR [5,6]. The most common contraindications to TPVI are: too small patients (<20–25 kg), too large RVOT (>30 mm), concomitant surgical maneuvers needed, RVOT gradient and right ventricular volumes below the cutoff values [5]. The majority of patients with clinical indications for RVOT intervention have large, compliant, noncircumferential outflow tracts previously modified by surgical transannular patch, surgical commissurotomy and annular incision/dilatation, or by catheter-based balloon valvuloplasty [7]. However, there is a progressive improvement in TPVI feasibility, thanks to the recent availability of larger devices, able to cover RVOT diameters >30 mm and a more accurate anatomical assessment by using 3D reconstruction and augmenter reality tools [[8], [9], [10], [11], [12]]. On the other hand, the surgical techniques in the past three decades were addressed to a pulmonary valve preservation and to a less generous RVOT enlargement by patch. Thus, the anatomical features of these patients are continuously changing, and is possible that the ratio of patients eligible for TPVI will be progressively higher [7,13].

The objective of this study was to conduct a survey from a sample of physicians from Italian implanting and non-implanting cardiological centers with a range of experience in PVR. Our aim was to gather information regarding current PVR practice, with a focus on the transcatheter approach, in particular: (1) patient selection and reasons for concern about TPVI, (2) patient management strategy, including pre-screening protocol and follow-up approaches, and (3) desired developments in the field.

## 2 Methods

A multidisciplinary Advisory Board (AB), made up of ten disease and therapy experts (4 from North-West, Milan – S. Donato Hospital; 3 from North-East, Padua University; and 3 from Middle-South, Bambino Gesù Pediatric Hospital - Rome) from the three main Italian centers of reference for congenital heart disease (CHD) treatment, was gathered with the responsibility of (1) developing specific questionnaires on PVR for CHD patients, with a focus on TPVI, (2) identifying and sending the questionnaires to physicians performing or referring patients for PVR, and (3) reviewing and interpreting the analyses. A four-steps approach was implemented. Details were reported in *Supplementary Data A.*

In the current guidelines, PVR and TPVI are indicated in symptomatic patients with severe pulmonary regurgitation (PR) and/or pulmonary stenosis (PS). Table 1 reports the identified recommendations for PVR in asymptomatic patients.

**Table 1:** Recommendations for PVR in asymptomatic patients from Published Guidelines of the American Heart Association/American College of Cardiology (AHA/ACC) and European Society of Cardiology (ESC).

Parameter	2021 ESC [2]	2018 AHA/ACC [14]
RV end-diastolic volume index [ml/m2]	≥160	≥160
RV end-systolic volume index [ml/m2]	≥80	≥80
RV dysfunction	Progressive RV dysfunction	≥ Mild
RVOT obstruction	RV systolic pressure >80 mmHg	RV systolic pressure ≥2/3 systemic pressure
PS	Severe	Not specified
PR	Severe	Moderate or severe
QRS duration [ms]	>180	Not specified
Exercise capacity	Objective decrease	Progressive reduction in objective exercise tolerance
Tricuspid Regurgitation	Progressive (at least moderate)	Not specified

Note: AHA/ACC guidelines require moderate or severe pulmonary regurgitation and, at least, 2 of the listed criteria. ESC guidelines require severe pulmonary regurgitation and/or stenosis and, at least, 1 of the listed criteria.

The survey was addressed to, and adequately tailored on, three different cluster of physicians:

• *Implanters (Group A)*: physicians working in implanting cardiological centers and performing TPVI procedures;
• *Clinicians from Implanting Centers (Group B)*: physicians working in implanting cardiological centers, but non performing TPVI procedures (e.g., heart-team members involved in patients screening for TPVI);
• *Clinicians from Non-Implanting Centers (Group C)*: physicians working in non-implanting cardiological centers and responsible for referring patients to specialist implanting centers.

The surveys consisted of 5 introductory questions aimed at stratifying the sample of respondents, and additional specific questions (11 for *Group A and C (2)* surveys and 8 for *Group B* survey, respectively) on clinical practice and potential improvements (survey questions in full are provided in the Supplementary material A).

Each AB member was asked to send the on-line surveys to at least 10 physicians from implanting centers located in their geographical area (North-West; North-East and Middle-South), as well as additional physicians from non-implanting centers, with a 4-week deadline for completion and a weekly reminder. The mailing list was built by the AB, based on personal and Institutional mailing list. A statement of consent was included at the start of the survey and consent was implied by completion and submission of the online survey.

### 2.1 Statistical analysis

Descriptive statistics were used to summarise respondents’ characteristics and survey results. This includes fractions and percentages for categorical variables and median (Q1-Q3) for continuous variables. No imputation was used. A two-sided p-value of less than 0.05 was considered to indicate statistical significance. All statistical analysis was performed using R Statistical Software (version 3.4.2; R Foundation for Statistical Computing, Vienna, Austria).

## 3 Results

### 3.1 Physicians surveyed

The physician involved in the survey were cardiologists, pediatricians, cardiac surgeons, experts in cardiac imaging who routinely manage CHDs in their in-patient or outpatient clinics. The surveys were sent to 454 mail addresses in total and 82 Italian physicians completed them anonymously via Qualtrics electronic platform [15] (response rate 18%). However, a possible overlap among the Group C physicians should be considered (i.e., some physicians could have been contacted twice or more by different AB members), thus increasing the real response rate.

**Fig. 1:**
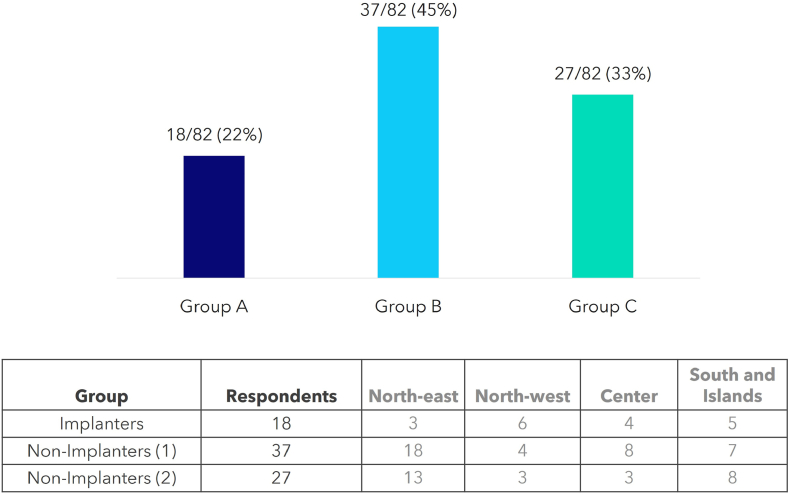
Summary of respondents by group and region.

The group and territorial distribution of those surveyed is displayed in Fig. 1.

More than half of respondents (46/82, 56%) were clinical cardiologists/pediatric cardiologists, 17% were interventional cardiologists (14/82), 13% were imaging experts (11/82), and 11 were cardiac surgeons (13%).

Nearly half of the answers came from physicians with at least 11 years of experience in the management of congenital patients (15% with 11–15 years of experience and 34% with >15 years of experience, respectively). The majority of respondents (61/82, 74%) currently manages both adult and pediatric congenital patients.

Based on implanters’ survey (*Group A*), the median number of annually performed TPVI procedures at their centers was >20 for a third of respondents (6/18, 33%), while the median number of annually performed surgical PVR was >10 for more than half of respondents (12/18, 67%).

These numbers were consistent with those reported by the other groups, with nearly 40% (15/37) of surveyed *Group B* managing between 10 and 20 patients eligible for PVR on average per year, and most of surveyed *Group C* (16/27, 59%) following less than 5 patients potentially eligible for PVR on average per year.

The main reported advantages of TPVI over surgical approach were reduced length of hospital stay (94% of *Group A*, 87% of *Group B*, and 59% of *Group C* respondents) and higher patients’ acceptance (89%, 84%, and 74%, respectively). On the other hand, the most substantial barriers to greater adoption of TPVI compared to surgical approach were the need of adequate anatomical characteristics (78%, 73%, and 56%), and the required skilled operators (39%, 43%, and 56%, respectively), due to its perceived complexity.

Interestingly, AB members noted that TPVI was considered as a “simple procedure” by just 4 out of 18 Implanters, thus confirming that it is not commonly perceived as an easier approach compared to the surgical PVR.

### 3.2 Patients’ management process

**Fig. 2:**
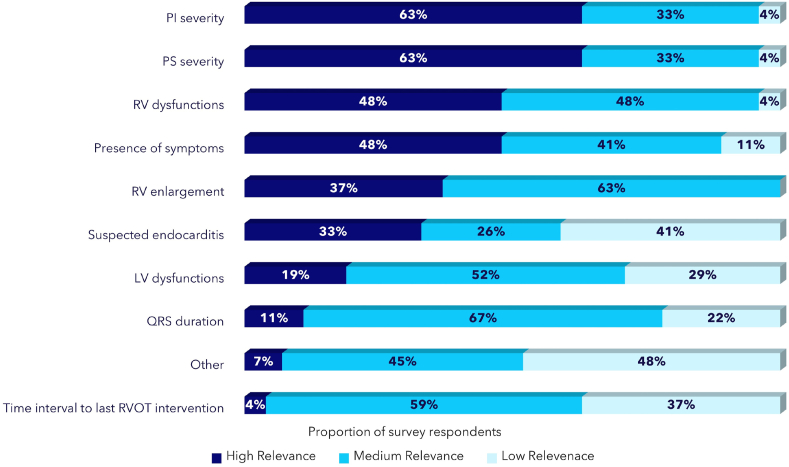
Parameters considered for referring patients for PVR - *Group C* survey PI: Pulmonary Insufficiency; PS: Pulmonary Stenosis; RV: Right Ventricle; LV: Left Ventricle; RVOT: Right Ventricular Outflow Tract.

**Fig. 3:**
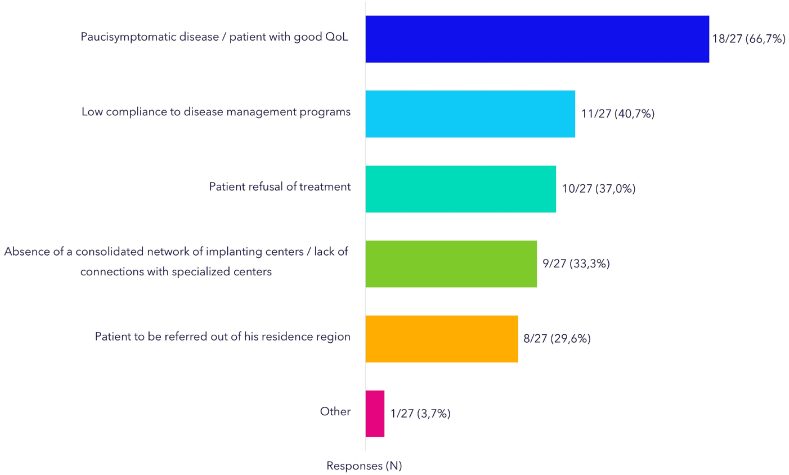
Factors influencing delayed referral of PVR patients - *Group C* survey QoL: Quality of Life.

According to the *Group C* answers, CHD patients followed by non-implanting cardiological centers usually undergo examinations by 2D/3D Echo Color Doppler (24/27, 89%), electrocardiogram (21/27, 78%) and/or by cardiac magnetic resonance imaging (MRI; 20/27, 74%) before being referred for PVR. The three most frequently selected parameters driving their decision of referring patients for PVR included (1) pulmonary insufficiency (PI) severity (63% first rank), (2) PS severity (63% first rank), (3) RV dysfunction (48% first rank) and (4) presence of symptoms (48% first rank) (Fig. 2), which seems to be in line with current Guideline recommendations [2,14]. Notably, 82% of *Group C* physicians refer patients for PVR without a preliminary evaluation on the type of the intervention (i.e., transcatheter versus surgery), but let the tertiary center as totally in charge of the decision (see also Supplementary material C and D). The main factors contributing to delays referral of patients to tertiary centers were: paucisymptomatic disease (18/27, 67%) and patients’ poor adherence to disease management programs (11/27, 41%). AB members also considered a reason for concern that *Group C* also indicated the “absence of a consolidated network of implanting centers/lack of connections with specialized centers” (33%) and “the need of referring the patient outside the region of residence” (30%) as factors potentially delaying the referral of patients to PVR (Fig. 3).

**Fig. 4:**
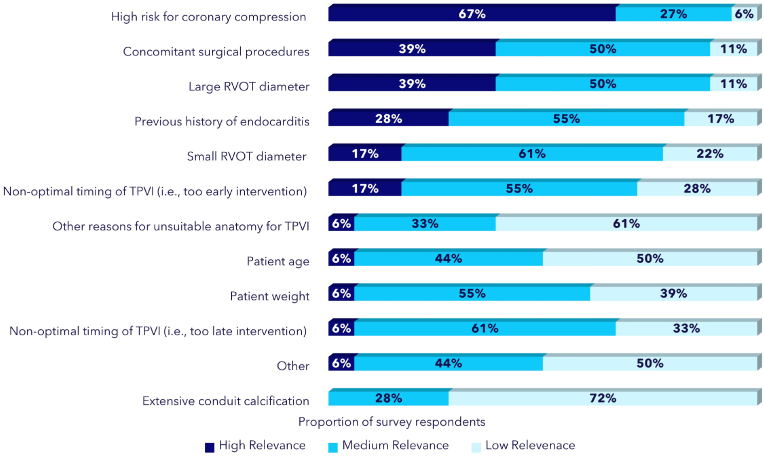
Main reported reasons leading respondent to refrain from TPVI - *Group A* survey RVOT: Right Ventricular Outflow Tract.

*Group A* considered high risk for coronary compression (67% first rank), the need for concomitant cardiac surgical procedures (39% first rank) and the unsuitable anatomy of the conduit (i.e., native RVOT with large diameter) (39% first rank) as the main reasons for refraining from TPVI. Other reported reasons included (1) a known previous history of endocarditis, (2) a small conduit diameter, or (3) non-optimal timing of TPVI (i.e., too early intervention) (Fig. 4). There was a higher preference of *Group A* for the cardiac computed tomography (CT) compared to clinicians of *Group B* (78% versus 70%).

### 3.3 Indications to TPVI

**Fig. 5:**
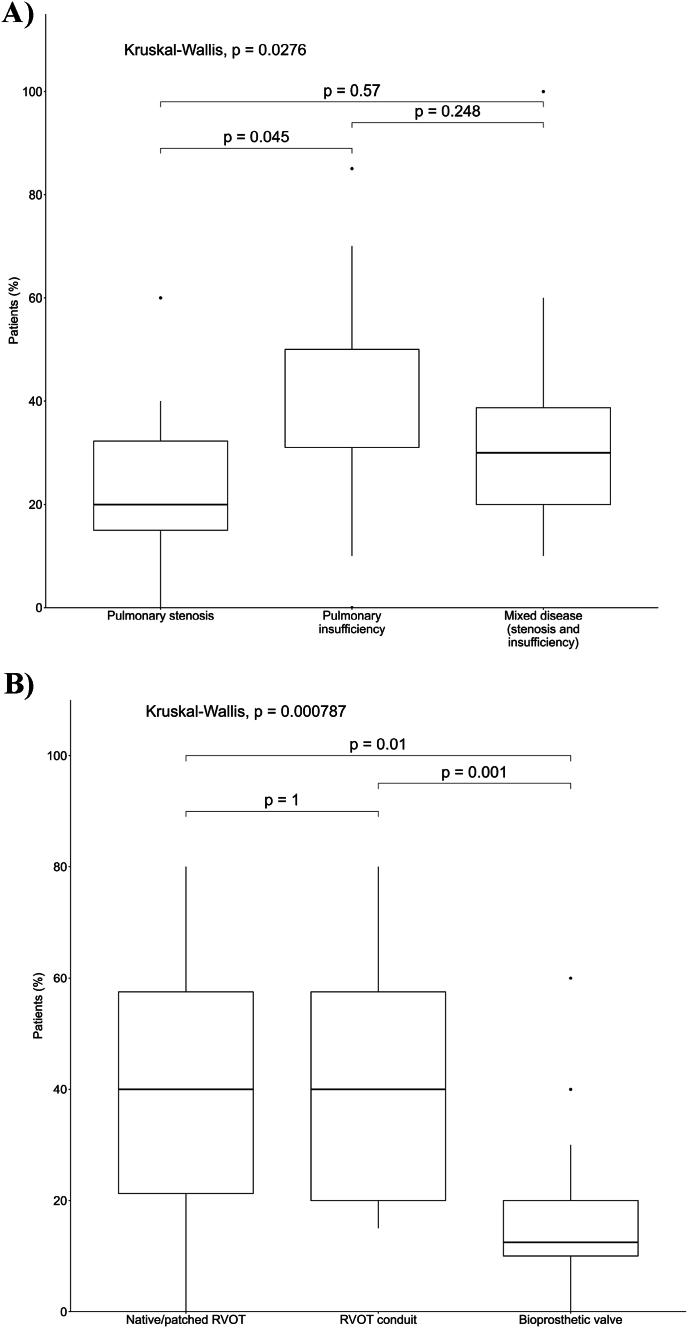
TPVI implanted patients' distribution - *Group A* survey. A) reports the Kruskal-Wallis test results on TPVI-treated patients distribution, according to their primary indication for the intervention, resulting in a significative difference between PI and PS implanted patients (p < 0.05). In boxplot charts, boxes indicate the interquartile range (IQR with the middle line corresponding to the median. Whiskers extend from each box to the farthest point within ±1.5 IQR. B) reports the Kruskal-Wallis test results on TPVI-treated patients distribution, according to their conduit type before the intervention, resulting in a significative difference between RVOT conduit and bioprosthetic valve patients (p = 0.001) and between native/patched RVOT and bioprosthetic valve patients (p = 0.01).

*Groups A and B* reported a prevalence of PI as primary RVOT dysfunction among the patients they treated by TPVI or that are referred to their centers as eligible for TPVI. Concerning the RVOT type, the majority of TPVI- treated or referred patients presented native/patched RVOT or RVOT conduit as anatomy before the valve replacement (Fig. 5).

These data are confirmed by *Group C* physicians, reporting that most of patients they refer to specialists for PVR presents PI, compared to PS or mixed disease, and has native/patched RVOT anatomy.

### 3.4 Follow-up

After the PVR procedure, most *Group C* physicians (63%) take care of patients’ follow-up management. For the remaining cases, patients are directly followed by the tertiary center performing the implant (33%) or follow-up visits are shared between tertiary and non-implanting centers (4%).

Among those who directly manage the follow-up care, all surveyed physicians (100%) stressed the value of 2D/3D Echo Color Doppler and electrocardiogram as routine exams for patients after valve replacement (generally every 6–12 months). Most of them (94%) also performs/prescribes Holter ECG monitoring as follow-up examination on a periodic basis.

### 3.5 Future perspectives

Finally, the *Group A* survey explored the respondents opinions regarding the priorities for future development within TPVI. The most selected technological development was new larger valves of a self-expandable nature to expand the cohort of patients currently eligible for TPVI (i.e., enabling TPVI in patients with large native RVOTs).

## 4 Discussion

The first TPVI in humans was performed in 2000 by Philipp Bonhoeffer. Since then, there was a progressively larger availability of tools and devices to broaden the feasibility of the *trans*-catheter approach. To date, in Europe, the devices available can cover RVOT diameters from 14 to 34 mm. In North America, since 2021, were already available two more devices (Harmony®, Medtronic; Alterra®, Edwards) able to cover RVOT diameters up to 40 mm^9,10^. Being the portfolio of devices for PVR continuously changing, the operators should be continuously updated on the tools available, on the specific indications and on the technical skills of each device. On the other hand, the indication to PVR reported in the guidelines [2,14] are still based on “old” studies, where the feasibility of TPVI was far lower than surgery [16].

Assenza et al. [17] compared AHA vs ESC guidelines on adult CHD (ACHD) patients. Basically, regarding RVOT lesions, the North American approach was based on an extended review of the available literature. Based on the data available up to the 2018, they reported a substantial equivalence in terms of costs and risk-to-benefit ratio between surgery and percutaneous approach. On the other hand, European guidelines were mainly based on experts’ opinion, and indicated a preference toward the percutaneous approach, when feasible. Most importantly, a large heterogeneity can be found between the cut-off values for PVR, either in stenosis or in regurgitation settings.

In summary, the guidelines on CHD patients are published with an interval of 5 years, and the studies cited are even older. On the other hand, the growing experience with new devices can widen the eligibility of patients to TPVI. In addition, the low procedural risk can suggest to anticipate the PVR in order to reduce the symptoms, increase the exercise capacity and to avoid the onset of arrhythmias and sudden events.

The choice of the right device at the right time for a given patient is not easy. In addition, many variables should be taken in account during the procedure. Probably, this is the reason why the implanters classified this procedure as challenging. This perception was also evident in the B and C groups. Thus, the need of dedicated and expert operators seems to be transversal.

On the other hand, the challenges of the procedure seem to be differently perceived from the 3 groups. Despite all the groups considered the procedure more accepted from patients than surgery, the difference between surgery and percutaneous approach were differently weighed form group C physicians. In summary, the *Group C* considers the TPVI a less accurate option compared to surgery, since the benefit of a percutaneous procedure is counterbalanced by a shorter durability and a limited feasibility. However, this perception was refused by recent studies who compared TPVI to surgery [[18], [19], [20], [21], [22]].

The real feasibility rate of TPVI is difficult to be assessed. If we consider all the patients requiring a PVR, TPVI was feasible in about 25% of them [6]. However, if we exclude infants and children, the feasibility rate is above the 60%. In addition, if we focus on the patients screened to perform the interventional procedures only, the feasibility rate raised above the 80%, despite challenging anatomies [23,24]. In this survey, we didn't explore the potential role of new non-invasive imaging options, like 3D printing, augmented reality and Metaverse. However, the possibility to check the feasibility of a procedure before proposing that procedure to the patient, as well as to show the intended procedure to the patient by using these new visual rendering can improve the adherence to the proposed care and reduce the percentage of unsuccessful procedures. Board members noted a higher preference of cardiac CT compared to non-implanters (i.e., B and C groups)*.* CT is fast, is an excellent tool to evaluate RVOT and vessels anatomy, including coronary arteries and pulmonary annulus diameter and circumference; in addition, CT is the gold standard to study RVOTs and conduits. Finally, CT images can be easily manipulated to build 3D models. On the other hand, B and C groups seem to prefer MRI in order to obtain functional data on right ventricular function and volumes and pulmonary valve regurgitation volumes.

The wondering of the right time for the procedure is a key-point. A timely PVR allows the recovery of right ventricular volume and function, improves the exercise capacity and the quality of life, and reduces the risk of sudden events [25]. On the other hand, the real impact of the residual defects on the daily activity of CHD patients is usually underestimated [26]. Thus, to wait until the onset of symptoms or a tangible effect on the quality of life of these patients may be too late. Recently, Bokma et al. [27] published the data from INDICATOR cohort (International Multicenter TOF Registry). They demonstrated that PVR was associated with a lower hazard of a composite endpoint of death or sustained ventricular tachycardia, and that the benefit of PVR was mainly apparent in patients with more advanced RV volume overload [27]. Despite all, 66.7% of the *Group C* physicians stated to not address the patients to PVR because of a good quality of life. In addition, in about 40% of the cases, the “conservative strategy” was driven by the poor compliance of the patients to an interventional procedure, despite the same physicians considered this procedure “more accepted from the patient” in a previous query. Finally, the lack of an official hospital network for the management of ACHD (33.3%), or a too far referral center (29.6%), were reported as reasons of a late presentation. The last point seems to be very important, because underlines how these patients are managed in outpatients’ clinics and in first level hospital, without a structured hub and spoke network. This finding may also suggest the confusion among the non-implanter centers in identifying a specialized ACHD referral center, highlighting the difficulty that a ACHD patient may face in Italy. Indeed, this result should encourage Italian scientific societies and public health care organization to improve the organization of service for the ACHD patients, a growing population. This point seems to be paradoxical, and likely underestimated, because the mail address of the participants was obtained from mailing lists of the referral centers, witnessing an already existing contact between the spoke center and at least one hub center.

Lastly, the questions addressed to evaluate the adherence of the clinical management, the appropriate pre-operative and post-operative work-up and the follow-up of these patient to the current guidelines showed a high level of appropriateness in all the three categories.

### 4.1 Limitations

The present survey reflects the experience of those physicians who choose to respond to our request and does not reflect the experience of all contacted physicians (18% of the e-mail sent). As for any anonymous survey based on voluntary participation, we can presume that willingness to participate may identify healthcare professionals with a specific interest on the topic and with specific knowledge on most recent technologies for TPVI, thus impacting the survey response rate. The mailing list used did not cover all the adult cardiology clinics available in Italy. Also, the survey questions did not investigate the patients’ perspective. Despite congenital heart disease are usually followed in a dedicated setting, we were not able to cover single physician that sporadically can face CHD patients.

## 5 Conclusion

TPVI is an emerging option for PVR. Despite many studies demonstrated the non-inferiority of TPVI compared to surgery and the availability of new devices for larger RVOT, the non-implanters perceive it as a niche procedure, to be reserved for symptomatic cases with a high surgical risk. Finally, the lack of an official hub-and-spoke network, a clear identification of ACHD referral center, limits access to care for a significant percentage of patients. Future studies should be addressed to identify clear cutoff and key parameter specific for TPVI, to get less ambiguous the indication to this procedure. In addition, formative events should be addressed to inform the non-implanters on the tools available for the patients’ selection and on the new devices able to cover larger RVOT sizes.

## Funding statement

All the Authors have received honoraria from Medtronic in relation to this project.

## Declaration of competing interest

The authors declare the following financial interests/personal relationships which may be considered as potential competing interests:Biagio Castaldi reports financial support and travel were provided by Medtronic. Gianfranco Butera reports financial support was provided by Medronic. Massimo Chessa reports financial support was provided by Medtronic. Lorenzo Galletti reports financial support was provided by Medtronic. Alessandro Giamberti reports financial support was provided by Medtronic. Luca Giugno reports financial support was provided by Medtronic. Aurelio Secinaro reports financial support was provided by Medtronic. Vladimiro Vida reports financial support was provided by Medtronic. Giovanni Di Salvo reports financial support was provided by Medtronic. Mario Carminati reports financial support was provided by Medtronic.
