# Supplementary material for: Transcatheter pulmonary valve implantation in clinical practice: A nationwide survey of cardiological implanting and non-implanting physicians
Source: Int J Cardiol Congenit Heart Dis. 2023 Oct 5;14:100478. doi: 10.1016/j.ijcchd.2023.100478 (PMC11658437; doi:10.1016/j.ijcchd.2023.100478)
Supplement: Multimedia component 1 [file mmc1.docx]

**Supplementary material**

**Supplementary Data A:**

**Four steps approach to build the survey and to manage the data obtained.**

**Step 1:** A literature review on recent evidence of TPVI for CHD patients with RVOT dysfunction was conducted. PubMed and EMBASE databases were searched from January 2011 to December 2021 using the search strategies: *('congenital heart disease') AND ('heart right ventricle outflow tract' OR 'heart right ventricle outflow tract obstruction' OR rvot OR 'right ventricular outflow tract' OR ('right ventricular' NEAR/3 (dysfunction* OR disease* OR obstruct*)) AND (((percutaneous OR transcatheter* OR 'trans catheter*') NEAR/5 ('pulmonary valve' OR 'pulmonary valves' OR 'pulmonic valve*')) OR 'transcatheter pulmonary valve implantation' OR 'pulmonary valve prosthesis)*. This resulted in 132 abstracts. After an in-depth review of the abstracts and full texts, a final number of 68 papers and 4 guidelines reporting information on patient selection criteria and outcomes of TPVI for CHD patients with RVOT dysfunction were selected and summarized as pre-reading material. The included papers were selected based on study type (non-systematic reviews, books, comments, abstracts, letters, and editorials were excluded), sample size (papers containing less than 10 patients analysed were excluded), and main outcomes of safety and/or effectiveness reported. Non-human studies and non-English publications were not considered for inclusion.

In the current guidelines, PVR and TPVI are indicated in symptomatic patients with severe pulmonary regurgitation (PR) and/or pulmonary stenosis (PS). Table 1 reports the identified recommendations for PVR in asymptomatic patients.

**Step 2.** The results of the literature review were discussed by the board members in a face-to-face meeting held in October 2022. Based on the discussion, three surveys, each specific for one of the following groups of respondents, were designed and finalized:

- *Survey Implanters (Group A)*: targeted at physicians working in implanting cardiological centers and performing TPVI procedures;
- *Survey Clinicians from Implanting Centers (Group B)*: targeted at physicians working in implanting cardiological centers, but non performing TPVI procedures (e.g., heart-team members involved in patients screening for TPVI);
- *Survey Clinician from Non-Implanting Centers (Group C)*: targeted at physicians working in non-implanting cardiological centers and responsible for referring patients to specialist implanting centers.

The surveys consisted of 5 introductory questions aimed at stratifying the sample of respondents, and additional specific questions (11 for *Group A and C* *(2)* surveys and 8 for *Group B* survey, respectively) on clinical practice and potential improvements (survey questions in full are provided in the ***Supplementary material A***).

**Step 3**. Each AB member was asked to send the on-line surveys to at least 10 physicians from implanting centers located in their geographical area (North-Ovest; North-East and Middle-South), as well as additional physicians from non-implanting centers, with a 4-week deadline for completion and a weekly reminder. The mailing list was built by the AB, based on personal and Institutional mailing list. The physician involved in the survey were cardiologists, pediatricians, cardiac surgeons, experts in cardiac imaging who routinely manage CHDs in their in-patient or outpatient clinics. The surveys were sent to 454 mail addresses in total and 82 Italian physicians completed them anonymously via Qualtrics electronic platform^15^ (response rate 18.2%). A statement of consent was included at the start of the survey and consent was implied by completion and submission of the online survey.

**Step 4.** A second face-to-face meeting was held in December 2022 to present and discuss the results of the surveys based on external physicians’ responses.

**Supplementary Data B:**

**Questions reported in the survey**

**Part 1: baseline questions (common to all the three groups)**

**Introduction**

1. In which geographical area do you mainly carry out your professional activity?

- North-east
- North-west
- Center
- South and Islands

1. What is your main specialty?

- Cardiologist
- Pediatric Cardiologist
- Cardiac surgeon
- Congenital cardiac surgeon
- Interventional physicians
- Imaging expert

1. Years of experience in the treatment / management of CHD patients:

- <5 years
- 5-10 years
- 11-15 years
- >15 years

1. I currently treat / manage ___ CHD patients:

- Adult
- Pediatric
- Both adult and pediatric

1. Are Transcatheter Pulmonary Valve Implant (TPVI) procedures performed at your center?

- Yes, I personally perform TPVI procedures 🡪 survey implanters (Group A)
- Yes, but I don’t perform TPVI procedures 🡪 survey clinicians from implanting centers (Group B)
- No, I work in a non-implanting cardiological center 🡪 survey clinicians from non-implanting centers (Group C)

**Survey Implanters - Group A**

1. What is the average number of TPVI procedures performed per year in your center?

- <5
- 5-10
- 10-20
- >20

1. What is the average number of surgical Pulmonary Valve Replacement (PVR) procedures performed per year in your center?

- <5
- 5-10
- 10-20
- >20

1. In your opinion, what are the main **advantage(s)** of TPVI compared to surgical PVR? (*select all that apply*)

- Lower short-term morbidity and mortality rates
- Reduced length of hospital stay
- High procedural success
- Simplicity and ease of procedure
- Higher patients’ acceptance of procedure
- Other, *specify:­­­___*

1. In your opinion, what are the main **limit(s)** of TPVI compared to surgical PVR? (*select all that apply)*

- Higher long-term reintervention rate
- Higher costs
- Anatomical limitations / limited feasibility of the procedure
- Procedure complexity / need for experienced operators
- Other, *specify:___*

1. Does your centre have a Heart Team dedicated to CHD patients?

- No
- Yes 🡪 *If yes:*

Who are the members of Heart Team? *(select all that apply)*

- Cardiologist
- CHD cardiologist
- Cardiac surgeon
- CHD cardiac surgeon
- Interventional physician
- CHD interventional physician
- Electrophysiologist
- CHD electrophysiologist
- Vascular surgeon
- Imaging expert
- Anaesthesiologist
- Other, *specify:___*

1. At your center, is the evaluation between TPVI and surgical PVR treatment made by a multidisciplinary team?

- No
- Yes 🡪 *If yes:*

Who are the multidisciplinary team members? *(select all that apply)*

- The Heart Team members *(exclusive option)*
- Cardiologist
- CHD cardiologist
- Cardiac surgeon
- CHD cardiac surgeon
- Interventional physician
- CHD interventional physician
- Electrophysiologist
- CHD electrophysiologist
- Vascular surgeon
- Imaging expert
- Anaesthesiologist
- Other, *specify:___*

1. What percentage of your TPVI treated patients has the following indication for PVR? *(the total must be 100%)*

- Pulmonary stenosis:*__%*
- Pulmonary insufficiency:*__%*
- Mixed disease (stenosis and insufficiency):*_%*

1. What percentage of your TPVI treated patients has the following anatomy before the procedure? *(the total must be 100%)*

- Native/patched Right Ventricular Outflow Tract (RVOT):*_%*
- RVOT conduit:*__%*
- Bioprosthetic valve:*__%*

1. What routine examinations do you perform or prescribe to the **TPVI eligible** CHD patients referred to your center? *(select all that apply)*

- 2D/3D Echo Color Doppler
- Electrocardiogram
- ECG Holter
- Stress electrocardiogram
- Cardiopulmonary test
- Cardiac magnetic resonance imaging
- Cardiac CT
- Pre-implant cardiac catheterization
- Other, *specify:___*

1. What are the main reasons that could lead you to **refrain from TPVI**? *Rank selected options from 1 (little or no relevance) to 5 (high relevance) according to your preference*

- Concomitant surgical procedures
- High risk for coronary compression
- Small RVOT diameter
- Large RVOT diameter
- Extensive conduit calcification
- Other reasons for unsuitable anatomy for TPVI*, specify:___*
- Previous history of endocarditis
- Patient age*, specify:___*
- Patient weight, *specify:___*
- Non optimal timing of TPVI (i.e., too late intervention)
- Non optimal timing of TPVI (i.e., early intervention)
- Other, *specify:___*

1. In your opinion, what technological improvement(s) would allow to expand the cohort of patients currently eligible for TPVI?

- Balloon-expandable valves with largest maximum diameter
- Self-expandable valves
- Mixed technologies
- Infundibular reducer devices
- Introduction of custom-made valves

**Survey Non Implanters (1) - Group B**

1. What is the average number of CHD patients **potentially eligible** for Pulmonary Valve Replacement (PVR) procedure you follow per year?

- <10
- 10-20
- 20-30
- >30

1. In your opinion, what are the main **advantage(s)** of TPVI compared to surgical PVR? (*select all that apply*)

- Lower short-term morbidity and mortality rates
- Reduced length of hospital stay
- High procedural success
- Simplicity and ease of procedure
- Higher patients’ acceptance of procedure
- Other, *specify:­­­___*

1. In your opinion, what are the main **limit(s)** of TPVI compared to surgical PVR? (*select all that apply)*

- Higher long-term reintervention rate
- Higher costs
- Anatomical limitations / limited feasibility of the procedure
- Procedure complexity / need for experienced operators
- Other, *specify:___*

1. Does your centre have a Heart Team dedicated to CHD patients?

- No
- Yes 🡪 *If yes:*

Who are the members of Heart Team? *(select all that apply)*

- Cardiologist
- CHD cardiologist
- Cardiac surgeon
- CHD cardiac surgeon
- Interventional physician
- CHD interventional physician
- Electrophysiologist
- CHD electrophysiologist
- Vascular surgeon
- Imaging expert
- Anaesthesiologist
- Other, *specify:___*

1. At your center, is the evaluation between TPVI and surgical PVR treatment made by a multidisciplinary team?

- No
- Yes 🡪 *If yes:*

Who are the multidisciplinary team members? *(select all that apply)*

- The Heart Team members *(exclusive option)*
- Cardiologist
- CHD cardiologist
- Cardiac surgeon
- CHD cardiac surgeon
- Interventional physician
- CHD interventional physician
- Electrophysiologist
- CHD electrophysiologist
- Vascular surgeon
- Imaging expert
- Anaesthesiologist
- Other, *specify:___*

1. What percentage of patients referred to your center and eligible for TPVI has the following indication for the procedure? *(the total must be 100%)*

- Pulmonary stenosis:*__%*
- Pulmonary insufficiency:*__%*
- Mixed disease (stenosis and insufficiency):*__%*

1. What percentage of patients referred to your center and eligible for TPVI has the following anatomy before the procedure? *(the total must be 100%)*

- Native/patched Right Ventricular Outflow Tract (RVOT):*_%*
- RVOT conduit:*__%*
- Bioprosthetic valve:*__%*

1. What routine examinations are performed or prescribed to the **TPVI eligible** CHD patients referred to your center? *(select all that apply)*

- 2D/3D Echo Color Doppler
- Electrocardiogram
- ECG Holter
- Stress electrocardiogram
- Cardiopulmonary test
- Cardiac magnetic resonance imaging
- Cardiac CT
- Pre-implant cardiac catheterization
- Other, *specify:___*

**Survey Non Implanters (2) - Group C**

1. What is the average number of CHD patients **potentially eligible** for Pulmonary Valve Replacement (PVR) procedure you follow per year?

- <5
- 5-10
- 10-20
- 20-30
- >30

1. In your opinion, what are the main **advantage(s)** of TPVI compared to surgical PVR? (*select all that apply*)

- Lower short-term morbidity and mortality rates
- Reduced length of hospital stay
- High procedural success
- Simplicity and ease of procedure
- Higher patients’ acceptance of procedure
- Other, *specify:­­­___*

1. In your opinion, what are the main **limit(s)** of TPVI compared to surgical PVR? (*select all that apply)*

- Higher long-term reintervention rate
- Higher costs
- Anatomical limitations / limited feasibility of the procedure
- Procedure complexity / need for experienced operators
- Other, *specify:___*

1. What routine examinations do you perform or prescribe to your CHD patients (e.g., Tetralogy of Fallot patients) **before referring them to the implanting center for PVR procedure**? *(select all that apply)*

- 2D/3D Echo Color Doppler
- Electrocardiogram
- ECG Holter
- Stress electrocardiogram
- Cardiopulmonary test
- Cardiac magnetic resonance imaging
- Cardiac CT
- Other, *specify:___*

1. What parameter(s) do you consider when **referring** a patient to the implanting center for PVR procedure? *Rank selected options from 1 (little or no relevance) to 5 (high relevance) according to your preference*

- Presence of symptoms
- Time interval to last RVOT intervention
- Pulmonary stenosis severity
- Pulmonary insufficiency severity
- Right ventricle dysfunction
- Left ventricle dysfunction
- Right ventricle enlargement
- QRS duration
- Suspected endocarditis
- Other, *specify:___*

1. When you refer a patient for PVR procedure:

- I usually do a preliminary evaluation on the type of the intervention (i.e., TPVI vs surgery PVR) whereby I refer the patient to an interventional cardiologist or to a surgeon
- I let the implanting center as totally in charge of the decision on the type of the intervention (i.e., TPVI vs surgery PVR)

1. What percentage of patients you refer for PVR has the following indication for the procedure? *(the total must be 100%)*

- Pulmonary stenosis:*__%*
- Pulmonary insufficiency:*__%*
- Mixed disease (stenosis and insufficiency):*__%*

1. What percentage of patients you refer for PVR has the following anatomy before the procedure? *(the total must be 100%)*

- Native/patched Right Ventricular Outflow Tract (RVOT):*_%*
- RVOT conduit:*__%*
- Bioprosthetic valve:*__%*

1. Do you take care of patient’s follow-up management following the PVR procedure?

- No 🡪 *If no:*

15a. Please indicate the reason:

- Patient’s follow-up is managed by the tertiary center performing the PVR implant
- Patient’s follow-up is managed by the [general practitioner](https://context.reverso.net/traduzione/inglese-italiano/general+practitioner)
- Other (specify)
- Yes 🡪 *If yes:*

15b. What routine examinations do you perform or prescribe to your PVR-treated patients during their follow-up? *(select all that apply, for the selected options please indicate the frequency)*

- 2D/3D Echo Color Doppler, *frequency: just for specific medical query; every 6 months; every 12 months; every 24 months; every 36 months*
- Electrocardiogram, *frequency: just for specific medical query; every 6 months; every 12 months; every 24 months; every 36 months*
- ECG Holter, *frequency: just for specific medical query; every 6 months; every 12 months; every 24 months; every 36 months*
- Stress electrocardiogram, *frequency: just for specific medical query; every 6 months; every 12 months; every 24 months; every 36 months*
- Cardiopulmonary test, *frequency: just for specific medical query; every 6 months; every 12 months; every 24 months; every 36 months*
- Cardiac magnetic resonance imaging, *frequency: just for specific medical query; every 6 months; every 12 months; every 24 months; every 36 months*
- Cardiac CT, *frequency: just for specific medical query; every 6 months; every 12 months; every 24 months; every 36 months*
- Other, *specify and report the frequency: just for specific medical query; every 6 months; every 12 months; every 24 months; every 36 months*

1. In your opinion, what are the main factors contributing to **delays** referral of patient to the implant center for PVR? *(select up to 3 options)*

- Patient to be referred out of his residence region
- Patient refusal of treatment
- Low compliance to disease management programs
- Paucisymptomatic disease / patient with good quality of life (QoL)
- Absence of a consolidated network of implanting centers / lack of connections with specialized centers
- Other, *specify:___*

**Supplementary data C:**

**Heart Team – presence and composition**

All of the *Group A* (18/18, 100%) and an overwhelming majority (35/37, 95%) of *Group B* respondents work at cardiologic implant centers having a multidisciplinary Heart Team (HT) specialized in the management of CHD patients. Congenital cardiologists, cardiac surgeons and interventional physicians form the basis of nearly all the reported HTs. Besides them, imaging experts and anesthesiologists were, in most instances, reported by *Group A* and *Group B* as part of the specialist HTs. Accordingly, nearly all *Group A* (17/18, 94%) and *Group B* (34/37, 92%) reported that the evaluation between transcatheter and surgical treatment is made by a multidisciplinary team, usually made by the same specialists composing the HT.

| **Question** | **Group A** | **Group B** |
| --- | --- | --- |
| Does your centre have a Heart Team dedicated to CHD patients? | No: 0/18 (0%)  Yes: 18/18 (100%) | No: 2/37 (5%)  Yes: 35/37 (95%) |
| If Yes, who are the members of Heart Team? *(select all that apply)* | CHD cardiologist: 18/18 (100%)  CHD cardiac surgeon: 18/18 (100%)  CHD interventional physician: 16/18 (89%)  Imaging expert: 14/18 (79%)  Anaesthesiologist: 13/18 (72%)  Interventional physician: 7/18 (39%)  CHD electrophysiologist: 7/18 (39%)  Cardiologist: 5/18 (28%)  Cardiac surgeon: 5/18 (28%)  Electrophysiologist: 5/18 (28%)  Vascular surgeon: 0/18 (0%)  Other: 0/18 (0%) | CHD cardiologist: 34/35 (97%)  CHD interventional physician: 33/35 (94%)  CHD cardiac surgeon: 33/35 (94%)  Imaging expert: 30/35 (86%)  Anaesthesiologist: 28/35 (80%)  CHD electrophysiologist: 22/35 (63%)  Cardiologist: 17/35 (49%)  Cardiac surgeon: 13/35 (37%)  Interventional physician: 12/35 (34%)  Electrophysiologist: 10/35 (29%)  Vascular surgeon: 4/35 (11%)  Other: 1/35 (3%) |
| At your center, is the evaluation between TPVI and surgical PVR treatment made by a multidisciplinary team? | No: 1/18 (6%)  Yes: 17/18 (94%) | No: 3/37 (8%)  Yes: 34/37 (92%) |
| If Yes, Who are the multidisciplinary team members? | The Heart Team members: 16/17 (94%)  Other: 1/17 (6%) | The Heart Team members: 25/34 (74%)  Other: 9/34 (27%) |

**Supplementary Data D:**

**Routine examinations for TPVI eligible patients at implanting centers**

TPVI eligible patients referred to the implanting centers undergo examinations mainly by 2D/3D Echo Color Doppler (94% of *Group A*, 100% of *Group B* respondents), cardiac magnetic resonance imaging (94% *Group A*, 87% of *Group B*respondents) and/or by electrocardiogram (89% *Group A*, 97% of *Group B*respondents) to evaluate the suitability for the intervention and comprehensively assess the valve landing zone.

| **Question** | **Group A** | **Group B** |
| --- | --- | --- |
| What routine examinations do you/are performed or prescribed to the TPVI eligible CHD patients referred to your center? *(select all that apply)* | 2D/3D Echo Color Doppler: 17/18 (94%)  Cardiac magnetic resonance imaging: 17/18 (94%)  Electrocardiogram: 16/18 (89%)  ECG Holter: 15/18 (83%)  Cardiac CT: 14/18 (79%)  Cardiopulmonary test: 13/18 (72%)  Pre-implant cardiac catheterization: 6/18 (33%)  Stress electrocardiogram: 5/18 (28%)  Other: 0/18 (0%) | 2D/3D Echo Color Doppler: 37/37 (100%)  Electrocardiogram: 36/37 (97%)  Cardiac magnetic resonance imaging: 32/37 (87%)  Pre-implant cardiac catheterization: 28/37 (76%)  ECG Holter: 27/37 (73%)  Cardiac CT: 26/37 (70%)  Cardiopulmonary test: 26/37 (70%)  Stress electrocardiogram: 16/37 (43%)  Other: 0/37 (0%) |
